# Diagnostic performance of Prof. Valmed, ChatGPT-5 Thinking, and OpenEvidence in rheumatology: A comparative evaluation

**DOI:** 10.1007/s00296-025-06068-y

**Published:** 2026-01-10

**Authors:** Phillip Kremer, Emily Langballe, Isabell Haase, Jonathan Bamberger, Sebastian Kuhn, Martin Krusche, Johannes Knitza

**Affiliations:** 1 Division of Rheumatology and Systemic Inflammatory Diseases, III. Department of Medicine, University Medical Center Hamburg-Eppendorf, Hamburg, Germany; 2 Department of Rheumatology, Institute of Clinical Medicine, Oslo University Hospital, University of Oslo, Oslo, Norway; 3 Institute of Clinical Medicine, University of Oslo, Oslo, Norway; 4 Institute for Digital Medicine, Philipps-Universität Marburg, University Hospital of Giessen and Marburg, Baldingerstrasse, Marburg, Germany

**Keywords:** ChatGPT, Large language models, Artificial intelligence, OpenEvidence, Retrieval-augmented generation

## Abstract

To compare the diagnostic performance of a subscription-based medical large language model (LLM) certified as a medical device (Prof. Valmed), a subscription-based general-purpose LLM (ChatGPT-5 Thinking), and a freely accessible medical LLM (OpenEvidence). Sixty vignettes covering rare rheumatic diseases and differential diagnoses were entered using a standardized prompt to generate five top diagnoses and respective diagnostic probabilities. Blinded rheumatologists categorized suggested diagnoses as identical, plausible, or diagnostically different. Diagnostic accuracy was assessed using proportions of identical and plausible diagnoses and a total diagnostic score. Analyses were descriptive. Group differences for the proportion of identical top diagnoses were explored using Cochran’s Q test and post-hoc McNemar tests. Processing time was recorded. OpenEvidence produced the highest proportion of identical top diagnoses (35.0%), followed by ChatGPT-5 Thinking (26.7%) and Prof. Valmed (23.3%); however, pairwise differences were not statistically significant. ChatGPT-5 Thinking achieved the highest total diagnostic score (226), followed by OpenEvidence (221) and Prof. Valmed (212). All systems showed markedly higher diagnostic probabilities for identical versus different diagnoses. Mean processing times ranged from 20 to 36 s. All three systems demonstrated broadly comparable diagnostic accuracy and processing times. Further benchmarking that incorporates additional evaluation dimensions is essential to inform safe and effective use of LLM-based clinical support.

**Supplementary Information:**

The online version contains supplementary material available at 10.1007/s00296-025-06068-y.

## Introduction

Rheumatology is a diagnostically challenging specialty, characterized by rare and complex diseases that frequently present with multiple, often nonspecific symptoms. As a result, both rheumatologists and non-rheumatologists commonly encounter difficulties in establishing timely and accurate diagnoses [1, 2]. Diagnostic delay is therefore common in rheumatology, particularly for rare diseases, and can result in avoidable morbidity by postponing effective treatment and prolonging exposure to ineffective or unnecessary therapies [3, 4].

Large language models (LLMs) are emerging as powerful supportive tools in clinical medicine, including rheumatology [5–10]. Their ability to synthesize vast amounts of knowledge and provide personalized answers to natural-language clinical questions promises to accelerate decision support in ways that traditional resources cannot [11]. This shift has sparked growing interest and surveys demonstrated that rheumatic patients and rheumatologists both agree that diagnostic decision support is the most relevant field of implementation in rheumatology for LLMs [6, 12]. Importantly, in a recent large national survey, the majority of patients stated that they would welcome their rheumatologists using AI for clinical decision support [12]. The swift integration of LLMs into clinical workflows has also led to the release of first official guidelines for their use in practice [11]. Krusche et al. demonstrated in 2023 that general-purpose LLMs, such as ChatGPT, can achieve diagnostic accuracy comparable to that of experienced rheumatologists [10]. Furthermore, benchmarking work has shown that general-purpose LLMs outperform traditional diagnostic decision support systems (DDSS), including long-established, certified medical products, in terms of diagnostic accuracy but importantly also speed [9].

Barriers to the clinical implementation of LLMs include their susceptibility to hallucinations, the absence of reliable references [13]. Medical domain-specific tools increasingly use LLMs to retrieve verified medical information and, through retrieval-augmented generation (RAG), translate this validated content into personalized answers, rather than relying solely on training data [8]. OpenEvidence, for example, is HIPAA-compliant and currently available free of charge to healthcare professionals after creating an account. It answers clinical questions using a retrieval-augmented generation (RAG) approach grounded in medical literature. Through collaborations with journals, it also provides access to otherwise paywalled content. In a small retrospective evaluation, physicians rated its answers highly in terms of clarity, clinical relevance, and evidence-based support [14]. Prof. Valmed, in contrast, is a subscription-based clinical decision support tool that emphasizes regulatory compliance and claims to be Europe’s first certified medical product approved for clinical use, thus having successfully overcome the regulatory complexities of LLMs as medical products [15]. Both systems aim to improve trustworthiness and safety in clinical use by embedding validated sources and verifiable references. These developments have expanded the landscape of LLM-based diagnostic tools, leaving clinicians with multiple options that differ in scope, cost, and governance. However, comparative evidence on whether such design and regulatory features translate into measurable diagnostic benefit is limited. In particular, head-to-head evaluations that include certified, RAG-enhanced medical products alongside general-purpose LLMs are lacking in rheumatology.

To address this gap, we conducted a comparative study of Prof. Valmed, ChatGPT-5 Thinking and OpenEvidence. We aimed to quantify and contrast their diagnostic accuracy, ranking behavior, probability calibration, and processing speed, and to test whether RAG enhancement and regulatory certification are associated with improved diagnostic performance in this controlled benchmarking setting.

## Methods

This study compared ChatGPT-5 Thinking, a general-purpose LLM, OpenEvidence, a free medical RAG-based LLM program, and Prof. Valmed, a subscription-based, RAG-based LLM program, certified as a class IIb medical device.

To ensure comparability of the results with a recent rheumatology benchmarking study of the diagnostic accuracy and usability of LLMs and traditional diagnostic decision support systems [9], the previously used methodology was adapted. In brief, anamnestic, symptom-based data, along with the patient’s age and gender of 60 representative vignettes encompassing rare rheumatic diseases and differential diagnoses were used. Fifty vignettes were sourced from high-impact journals and ten vignettes were created based on real-world clinical cases from the Department of Rheumatology at University Hospital Hamburg-Eppendorf, Germany. The same prompt (see supplementary file 1) containing the vignette information was sequentially input using the respective web interfaces between August 25 and August 27, 2025. A new session was created for each vignette to ensure that the models did not retain information from previous interactions that could influence their responses. Case processing time was measured. The LLMs were also instructed to provide a diagnostic probability for each suggested diagnosis. The suggested diagnoses were independently and blindly reviewed by three rheumatologists from three different university hospital centers. Diagnostic suggestions were classified by the raters as identical, plausible, or diagnostically different, according to the procedures described by Hautz et al. [16] and Bastakoti et al. [17]. The interrater agreement was very strong, with Cohen’s κ of 0.907 for top diagnoses and 0.846 for all diagnoses. In cases of disagreement, a fourth board-certified rheumatologist adjudicated the final categorization. This process yielded the proportions of identical and plausible diagnostic suggestions, serving as indicators of diagnostic accuracy. To further quantify diagnostic performance, a total diagnostic score, adapted from Goh et al. [18], was applied, assigning two points for identical and one point for plausible diagnoses. A formal ethics inquiry was submitted to the Ethics Committee of Philipps University Marburg, Germany (reference 24–221 ANZ). Given the anonymous and non-interventional study design, ethical review board approval was waived.

### Data analysis

Vignette characteristics and study outcomes were summarized using descriptive statistics. The total diagnostic score was computed by summing all assigned points per case. Frequencies and percentages of identical and plausible diagnoses were reported, along with mean diagnostic probabilities for all diagnoses within each diagnostic category (identical, plausible, and different). Differences in proportions of correct top diagnoses were analyzed using Cochran’s Q test. When Cochran’s Q indicated a significant overall difference, pairwise McNemar tests with Holm correction for multiple comparisons were performed to identify which systems differed from one another. All statistical tests were two-sided, and significance was defined as *p* < 0.05. Analyses were conducted using Python (version 3.12.7) with the libraries pandas (version 2.3.2), NumPy (version 2.3.3), SciPy (version 1.16.3), and statsmodels (version 0.14.5).

## Results

**Fig. 1:**
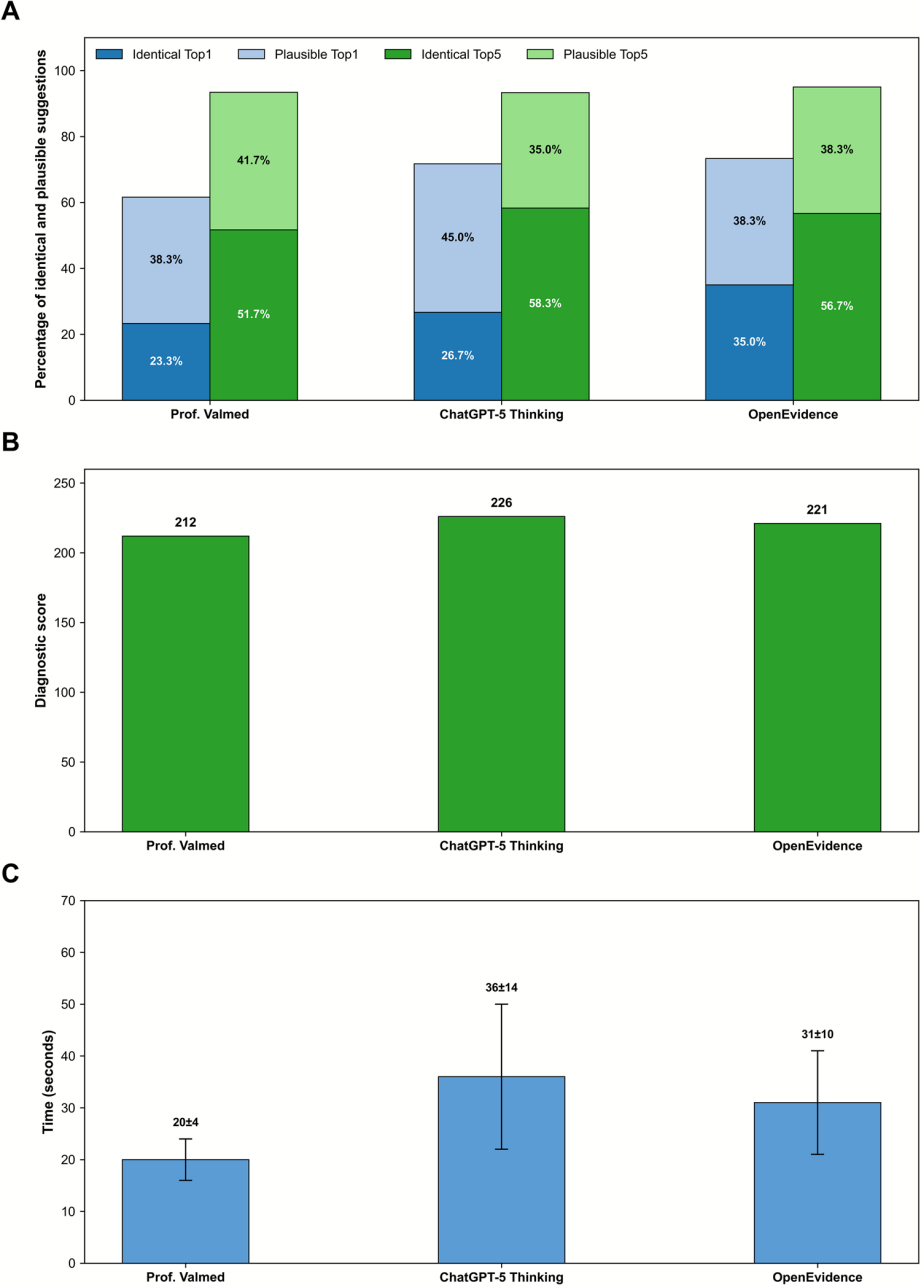
Percentage of vignettes with the identical (dark colours) or a plausible (light colours) diagnosis as the top suggestion (blue) and within the top 5 suggestions (green) (**A**), total diagnostic scores according to system (**B**), and mean (SD) case processing time (**C**)

**Fig. 2:**
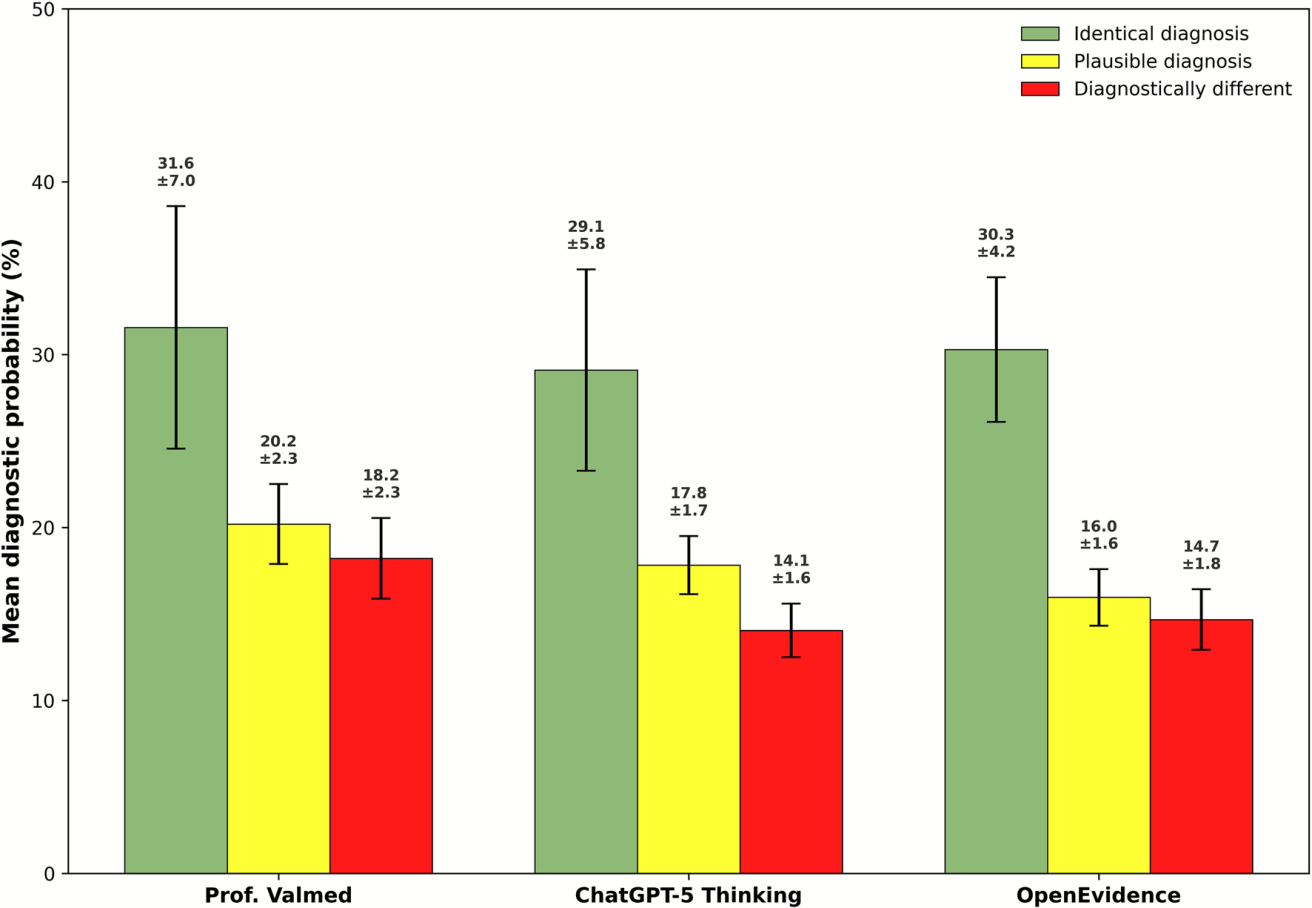
Mean diagnostic probabilities for all suggested diagnoses across identical, plausible and diagnostically different categories

Cochran’s Q test indicated a significant difference in diagnostic accuracy among the three systems (p = 0.001), however a post-hoc McNemar tests (Holm-adjusted) did not reveal significant differences between the individual systems (Prof. Valmed vs OpenEvidence: p = 0.0654; ChatGPT-5 Thinking vs OpenEvidence: p = 0.1797; Prof. Valmed vs ChatGPT-5 Thinking: p = 0.7905). OpenEvidence achieved the highest proportion of identical top diagnoses (Top1: 35.0%, Top5: 56.7%), followed by ChatGPT (Top1: 26.7%, Top5: 58.3%), and Prof. Valmed, (Top1: 23.3%, Top5: 51.7%), see Fig. 1A. ChatGPT achieved the highest total diagnostic score (226), followed by OpenEvidence (221) and Prof. Valmed (212), see Fig. 1B. Mean processing times were 20 s for Prof. Valmed, 36 s ChatGPT, and 31 s for OpenEvidence (Fig. 1C). Overall diagnostic accuracy varied substantially across models and individual cases (Supplementary Material 2). Mean diagnostic probabilities were comparable across all three systems, and each system reported markedly higher probabilities for identical diagnoses than for diagnostically different ones (Fig. 2).

## Discussion

This study is, to our knowledge, the first to compare the (diagnostic) performance of a ChatGPT model with Prof. Valmed, a certified, subscription-based RAG-enhanced LLM medical product, and OpenEvidence, a freely accessible, medical RAG-based system. While the overall frequency of identical or plausible suggestions did not differ substantially between systems, OpenEvidence provided a numerically higher proportion of identical top-ranked diagnoses. This distinction is important in practice, as clinicians generally rely on a focused set of leading suggestions and may disregard tools that return an overly broad range of possibilities [19]. The absence of a significant benefit for the two medical RAG systems aligns with a recent benchmarking study, underscoring how difficult it remains for RAG to yield robust clinical gains over general models, though that study also suggests ways to overcome key barriers [20]. RAG-augmented medical LLMs aim to boost factual accuracy by anchoring their responses to retrieved scientific sources. In rare rheumatic conditions, though, retrieval may break down because pertinent evidence is limited, hard to locate, or mismatched to how the clinical question is formulated [20–22]. And even when suitable documents are retrieved, the model may not reliably weave them into its answer, which can blunt any observable improvement in diagnostic performance.

Easy access, seemingly strong performance, and minimal costs will likely continue to drive clinicians toward using general-purpose, non-certified LLMs for diagnostic support [6]. However, the ethical principle of primum non nocere (“first, do no harm”) requires that patient safety remains paramount [23, 24]. This is precisely why regulatory guardrails exist, even when they introduce constraints that may modestly reduce benchmark performance. Medical-device certification is therefore designed to secure safe and reliable use through risk management, traceability, and rigorous quality assurance across the product lifecycle [25]. These processes can improve consistency, governance, and transparency, but they are not primarily intended to maximize performance. Comparing our findings with the previous comparative study, which evaluated the same set of cases, both OpenEvidence and Prof. Valmed demonstrated higher proportions of identical top diagnoses than the three traditional diagnostic decision support systems (Isabel DDx, Ada, Symptoma). However, their performance remained substantially lower than that of the four large language models analyzed, including Gemini, Llama, ChatGPT-4o, and Claude. Notably, ChatGPT-5 Thinking yielded a lower proportion of identical top diagnoses than ChatGPT-4o (26.7% vs. 40.0%), whereas their proportions of identical Top-5 diagnoses were similar (58.3% vs. 60.0%). This pattern aligns with previous observations that the performance of ChatGPT models may decline over time [26]. Importantly, all three LLMs evaluated in this study reported substantially larger probabilities for identical diagnoses than for diagnostically different ones, compared to the four previously analyzed LLMs. This finding is relevant for fostering clinician trust, particularly given that certified AI-based medical products such as Ada and Symptoma have previously been shown to assign higher probabilities to diagnostically different diagnoses than to identical ones [9, 27]. Another evaluation found that OpenEvidence provided significantly more accurate answers on skin malignancies compared to ChatGPT [28]. Similarly, a study by Low et al. using 50 clinical questions showed that special-purpose LLMs, including OpenEvidence, consistently outperformed general-purpose models in generating reliable answers [29]. These findings further align with a recent comparative study of 15 LLMs on osteoarticular infections, in which OpenEvidence achieved the highest overall score [30].

Overall usability differences between the systems included that ChatGPT-5 Thinking did not provide references. Prof. Valmed allows users to specify which reference sources should be queried (we selected all available sources), yet it frequently returned citations that appeared unrelated, often drawing from drug information sheets. In contrast, OpenEvidence consistently displayed relevant references and routinely included two additional “Not to Miss Diagnoses,” although these supplementary suggestions did not add any further identical or plausible diagnoses. Another difference, although not relevant to this study, is that Prof. Valmed and OpenEvidence currently rely exclusively on text input and do not support image-based analysis. Processing times for all systems averaged under one minute, making the delay clinically negligible.

To further enhance diagnostic accuracy and reduce the need for extensive manual data entry by clinicians, access to comprehensive patient data including integration with electronic health records would be essential [31]. Given the considerable heterogeneity in case-level performance (Supplementary Material 2) in line with previous studies [9, 30], combining the strengths of different models within a coordinated multi-agent diagnostic framework may represent a promising strategy to mitigate individual system limitations [32].

A potential limitation of this study is the possibility that LLMs may have been exposed to the previously published benchmarking vignette collection or the original published case reports. Such “data leakage” is an inherent benchmarking challenge and is difficult to exclude entirely. Although this risk can be reduced by withholding answers, paraphrasing vignettes, and regularly updating or regenerating benchmark datasets, residual exposure remains possible. We analyzed all references generated by the models in detail. In no instance did any model cite the earlier publication or the original case reports. However, the absence of explicit citations does not rule out such exposure, particularly for general-purpose LLMs. A single standardized prompt was intentionally used to preserve comparability with the benchmark methodology of Kremer et al. [9], however LLM outputs are sensitive to prompt phrasing and structure. Accordingly, results reflect performance under a single clinically motivated prompt and may not generalize across prompting strategies. Given the exploratory aim and lack of prior effect-size estimates, no a priori power calculation was done, so the study may be underpowered for small between-model differences. This also limits the generalizability of the findings and underscores the need for larger, more diverse case sets in future evaluations. Importantly, a 2025 comparative study found that, when evaluated against five general-purpose LLMs, OpenEvidence demonstrated the highest internal consistency, meaning that identical prompts reliably produced the same answers [33]. Such reproducibility is crucial for building physician trust, as inconsistent outputs may undermine clinical confidence in these tools. Internal consistency, however, was not assessed in the present study. Future benchmarking efforts should therefore evaluate not only diagnostic accuracy and processing speed but also internal consistency as acore quality indicator. While vignette-based studies provide a practical and controlled method for benchmarking diagnostic systems, real-world testing with end users, including patients and clinicians, remains essential to fully understand clinical utility and workflow integration. Finally, because this study represents only a snapshot of system performance at a single point in time, and given the rapid evolution of LLMs, periodic re-evaluation will be necessary to maintain an up-to-date understanding of their capabilities.

## Conclusion

This study shows that a subscription-based general-purpose LLM (ChatGPT-5 Thinking), a certified subscription-based medical LLM (Prof. Valmed), and a freely accessible medical LLM (OpenEvidence) demonstrated broadly comparable performance with respect to diagnostic accuracy, diagnostic probabilities, and processing time. Ongoing benchmarking across a wider range of evaluation domains will be essential to ensure safety and effectiveness as these systems continue to evolve.

## Supplementary Information

Below is the link to the electronic supplementary material.

Supplementary file1 (DOCX 55 KB)

Supplementary file2 (PDF 62 KB)

## Data Availability

The raw data supporting the conclusions of this article will be made available by the authors upon reasonable request.
