# Supplementary material for: Diagnostic performance of Prof. Valmed, ChatGPT-5 Thinking, and OpenEvidence in rheumatology: A comparative evaluation
Source: Rheumatol Int. 2026 Jan 10;46(1):31. doi: 10.1007/s00296-025-06068-y (PMC12790495; doi:10.1007/s00296-025-06068-y)

Prompt used in this study, based on Kremer P, Schiebisch H, Lechner F, *et al.* Comparative analysis of large language models and traditional diagnostic decision support systems for rare rheumatic disease identification. *EULAR Rheumatol Open*. 2025;1:51–9. doi: 10.1016/j.ero.2025.04.007


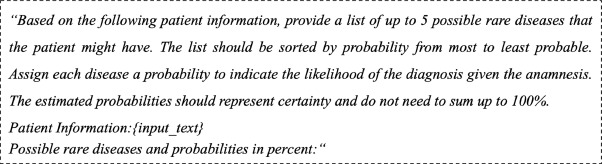

Supplement: Supplementary file 1 — Supplementary file1 (DOCX 55 KB) [file 296_2025_6068_MOESM1_ESM.docx]
