# Supplementary material for: Diagnostic performance of Prof. Valmed, ChatGPT-5 Thinking, and OpenEvidence in rheumatology: A comparative evaluation
Source: Rheumatol Int. 2026 Jan 10;46(1):31. doi: 10.1007/s00296-025-06068-y (PMC12790495; doi:10.1007/s00296-025-06068-y)
Supplement: Supplementary file 2 — Supplementary file2 (PDF 62 KB) [file 296_2025_6068_MOESM2_ESM.pdf]

Figure 1. Heat map displaying diagnostic accuracy of all vignettes according to respective system.

Patient	Diagnosis	Prof. Valmed	ChatGPT-5 Thinking	OpenEvidence
P1	VEXAS syndrome			
P2	Hereditary angioedema			
P3	Schnitzler syndrome			
P4	Amyotropic lateral sclerosis			
P5	Sjögren's disease with Cryoglobulinemia			
P6	Systemic lupus erythematosus			
P7	Fabry disease			
P8	Hemophagocytic lymphohistiocytosis			
P9	Eosinophilic granulomatosis with polyangiitis			
P10	Acute liver failiure			
P11	RAS-associated autoimmune leukoproliferative disease			
P12	Paroxysmal nocturnal hemoglobinuria			
P13	Hemochromatosis			
P14	Lead Intoxication			
P15	Dermatomyositis			
P16	Erdheim-Chester disease			
P17	IPEX syndrome			
P18	Susac syndrome			
P19	IgG4-related disease			
P20	Idiopathic multicentric Castleman disease			
P21	IgA-Vasculitis			
P22	Facioscapulohumeral muscular dystrophy			
P23	Thyreotoxic periodic paralysis			
P24	Relapsing polychondritis			
P25	Cogan syndrome			
P26	SAPHO syndrome			
P27	Degos disease			
P28	Limited cutaneous systemic sclerosis			
P29	Refsum disease			
P30	Common variable immunodefiency syndrome			
P31	Pompe disease			
P32	Behcet disease			
P33	Cryoglobulinemic vasculitis			
P34	Polyarteritis nodosa			
P35	Scurvy			
P36	Systemic lupus erythematosus with Anti-Phospholipid-Syndrome			
P37	Acut intermittent porphyria			
P38	Chronic granulomatous disease			
P39	Kaposi sarcoma			
P40	Systemic mastocytosis			
P41	Kikuchi-Fujimoto disease			
P42	Whipples disease			
P43	Hypereosinophilic syndrome with Löffler-Endocarditis			
P44	STING-associated vasculopathy with onset in infancy			
P45	Takayasu arteritis with subclavian steel syndrome			
P46	Scleroderma renal crisis			
P47	Mixed connective tissue disease			
P48	Anti-Synthetase syndrome			
P49	Drug-induced systemic lupus erythematosus			
P50	Dermatomyositis			
P51	Systemic sclerosis			
P52	Still's disease			
P53	Familial mediterranean fever			
P54	Granulomatosis with polyangitis			
P55	Mantelcell lymphoma with Hemophagocytic lymphohistiocytosis			
P56	VEXAS syndrome			
P57	Dermatomyositis			
P58	Chronic infantile neurological cutaneous and articular syndrome			
P59	Inclusion body myositis			
P60	Anti-Synthetase syndrome			

	No plausible or correct diagnosis mentioned
	Plausible diagnosis among top 5 suggestions
	Correct diagnosis among top 5 suggestions
	Correct diagnosis among top 1 suggestion
